# Special Issue “Machine Learning and Bioinformatics in Human Health and Disease 2nd Edition”

**DOI:** 10.3390/ijms27188005

**Published:** 2026-09-09

**Authors:** Andreas Leiherer

**Affiliations:** 1 Vorarlberg Institute for Vascular Investigation and Treatment (VIVIT), A-6800 Feldkirch, Austria; andreas.leiherer@vivit.at; 2 Central Medical Laboratories, A-6800 Feldkirch, Austria; 3 Faculty of Medical Sciences, Private University of the Principality of Liechtenstein, FL-9495 Triesen, Liechtenstein

## 1. Introduction

Machine learning and bioinformatics have become indispensable components of modern biomedical research [1]. The first edition of this Special Issue [2] highlighted the breadth of their applications, ranging from multi-omics integration and disease prediction to biomarker discovery and explainable artificial intelligence (AI).

The contributions to this second edition reflect the continuing maturation of the field. Computational approaches are becoming more closely integrated with biological research and are evaluated not only by their analytical performance, but also by their interpretability, robustness, and potential relevance for biomedical and clinical research. This development is particularly important as increasingly complex computational methods are applied to questions with direct biological and, in some cases, clinical implications.

This editorial discusses three aspects of this development: (i) the interpretation of molecular data in a mechanistic context, (ii) the integration of biological knowledge into computational models, and (iii) the growing importance of interpretability, robustness, and validation. Together, these aspects illustrate how computational and biological approaches are becoming increasingly interconnected in contemporary biomedical research.

## 2. From Molecular Signatures to Biological Mechanisms

Several contributions to this Special Issue illustrate how computational analyses can connect molecular alterations with their biological context and potential role in disease. By integrating different layers of molecular information, these studies provide insights into regulatory relationships, pathways, and mechanisms that may underlie disease development.

The individual contributions illustrate this evolution from complementary biological perspectives. Zhang and colleagues (Contribution 1) used complementary network-based approaches to characterize molecular pathways linking oxidative stress to pain–depression comorbidity. Their study identified biologically coherent pathways and regulatory networks that provide mechanistic hypotheses for disease development. Similarly, Xu and colleagues (Contribution 2) moved beyond differential expression analysis by reconstructing miRNA–mRNA regulatory networks associated with triple-negative breast cancer, thereby placing individual molecular alterations into their biological context. This transition is closely related to the concept of network medicine, which views diseases as perturbations of interconnected biological systems rather than isolated molecular events [3].

A comparable concept is evident in the work of De Filippis and colleagues (Contribution 3). Recent advances in computational prediction of missense variant effects have substantially expanded the possibilities for functional interpretation of genetic variation [4]. By integrating complementary computational resources, the authors transformed the analysis of more than 27,000 missense variants into biologically meaningful insights regarding evolutionary constraints, protein function, and nutrition-related pathways. Likewise, Heinzle and colleagues (Contribution 4) showed that oxaliplatin resistance is accompanied by distinct volatilomic alterations consistent with changes in oxidative stress and cellular metabolism.

Taken together, these studies illustrate how computational analyses can connect molecular signatures with the biological mechanisms underlying disease. This mechanistic perspective represents an important step in the continuing evolution of computational biomedicine.

## 3. Integrating Biological Knowledge into Computational Models

Existing biological knowledge can contribute to computational analyses in different ways. It may be incorporated directly into model construction [5] or provide a framework for the biological interpretation of data-driven results. The contributions presented in this Special Issue illustrate both strategies.

The scANMF framework developed by Chi and colleagues (Contribution 5) exemplifies the first strategy. Cell-type annotation remains one of the principal challenges in single-cell transcriptomics, particularly when datasets are affected by technical noise, incomplete reference atlases, or differences between sequencing platforms and species [6,7]. Instead of relying on a single source of information, the model integrates established marker genes, partial cell-type annotations, and graph-based representations of cellular relationships within a unified analytical framework. This combination improves annotation accuracy while preserving biological interpretability across independent datasets, sequencing platforms, and even different species, illustrating how multiple complementary sources of biological knowledge can enhance both model performance and robustness.

The second strategy is illustrated by the pharmacogenomic study of Brlek and colleagues (Contribution 6). Using unsupervised learning, the authors identified population subgroups that were subsequently linked to the well-established pharmacogenes CYP2D6 and SLCO1B1. The computationally identified patient clusters could thus be related to established pharmacogenomic knowledge relevant to drug metabolism and individualized therapy.

Although these studies address different biomedical questions using different computational methodologies, they illustrate the same conceptual development. Computational models are increasingly developed and interpreted in the context of existing biological knowledge. Computational methods and biological expertise therefore represent complementary sources of evidence that, together, can generate more robust and biologically meaningful conclusions.

Future progress in computational biomedicine will therefore depend on the successful integration of methodological innovation and biological knowledge.

## 4. From Accurate Predictions to Trustworthy Models

As computational models become increasingly integrated into biomedical research and clinical decision-making, the criteria by which they are evaluated are also changing. Predictive accuracy remains an essential benchmark, but it is no longer sufficient. Computational models are now expected to be transparent and interpretable [8,9,10], while robustness, biological plausibility, and independent validation provide additional evidence for their reliability. This evolution reflects a broader shift in computational biomedicine: confidence is earned not by performance metrics alone, but by the quality of evidence supporting computational conclusions.

Ultimately, clinical decisions must remain understandable not only to physicians but also to patients, particularly if AI is to strengthen rather than erode the physician–patient relationship [11]. If a computational model identifies a patient as being at high risk, clinicians should be able to explain the biological and clinical rationale rather than simply responding, “because the computer said so”. Understanding why a model reaches a particular conclusion may therefore facilitate its integration into clinical reasoning, improve communication between physicians and patients, and support more informed decisions.

The studies presented in this Special Issue illustrate this development in complementary ways. Though predictive performance is still important, the studies demonstrate that robustness, interpretability, and biological validation are integral components of recent high-quality computational research.

The scANMF framework developed by Chi and colleagues (Contribution 5) provides an instructive example. The authors demonstrated excellent annotation performance and systematically evaluated the robustness of their model across independent datasets, sequencing technologies, and species. They further examined the effects of incomplete annotations, noisy marker genes, and model components through extensive sensitivity and ablation analyses. Together, these analyses provide evidence for the robustness of scANMF under heterogeneous and imperfect data conditions.

A complementary perspective is provided by HazChemNet (Contribution 7). Although the deep-learning framework achieved excellent predictive performance for chemical hazard classification, the study deliberately extended model evaluation beyond conventional benchmarking. Feature ablation analyses identified the molecular characteristics contributing most strongly to prediction, while external validation demonstrated generalizability to previously unseen compounds. Most importantly, selected computational predictions were supported by experimental validation using Caenorhabditis elegans, thereby linking computational inference with traditional biological evidence.

Despite addressing different biomedical questions, both studies highlight the same principle. Confidence in computational models arises from the convergence of multiple forms of evidence. Robust validation, biological interpretability, external testing, and experimental confirmation each contribute to establishing trust in computational findings. Together, they transform predictive models into scientifically reliable tools.

As computational methods increasingly influence biological discovery and clinical research, methodological excellence alone will no longer be sufficient. Future computational models must combine high predictive performance with reproducibility, biological plausibility, and robust validation if they are to support scientific or clinical decision-making. Ultimately, trust is built through evidence—not through accuracy alone.

## 5. Concluding Remarks

The studies assembled in this Special Issue demonstrate that the future of computational biomedicine lies in combining methodological sophistication with biologically meaningful knowledge that researchers can validate, clinicians can trust, and patients can ultimately benefit from.
